# Multifunctional scaffolds for biomedical applications: Crafting versatile solutions with polycaprolactone enriched by graphene oxide

**DOI:** 10.1063/5.0184933

**Published:** 2024-03-01

**Authors:** G. Friggeri, I. Moretti, F. Amato, A. G. Marrani, F. Sciandra, S. G. Colombarolli, A. Vitali, S. Viscuso, A. Augello, L. Cui, G. Perini, M. De Spirito, M. Papi, V. Palmieri

**Affiliations:** 1 Dipartimento di Neuroscienze, Università Cattolica del Sacro Cuore, Largo Francesco Vito 1, 00168 Roma, Italy; 2 Fondazione Policlinico Universitario “A. Gemelli” IRCSS, Largo A. Gemelli 8, 00168 Roma, Italy; 3 Dipartimento di Chimica, Università di Roma “La Sapienza,” p.le A. Moro 5, I-00185 Roma, Italy; 4 Istituto di Scienze e Tecnologie Chimiche “Giulio Natta”-SCITEC (CNR), C/O Istituto di Biochimica e Biochimica Clinica, Università Cattolica del Sacro Cuore, L.go F. Vito 1, 00168-Roma, Italy; 5 Istituto dei Sistemi Complessi, CNR, via dei Taurini 19, 00185 Roma, Italy

## Abstract

The pressing need for multifunctional materials in medical settings encompasses a wide array of scenarios, necessitating specific tissue functionalities. A critical challenge is the occurrence of biofouling, particularly by contamination in surgical environments, a common cause of scaffolds impairment. Beyond the imperative to avoid infections, it is also essential to integrate scaffolds with living cells to allow for tissue regeneration, mediated by cell attachment. Here, we focus on the development of a versatile material for medical applications, driven by the diverse time-definite events after scaffold implantation. We investigate the potential of incorporating graphene oxide (GO) into polycaprolactone (PCL) and create a composite for 3D printing a scaffold with time-controlled antibacterial and anti-adhesive growth properties. Indeed, the as-produced PCL-GO scaffold displays a local hydrophobic effect, which is translated into a limitation of biological entities-attachment, including a diminished adhesion of bacteriophages and a reduction of *E. coli* and *S. aureus* adhesion of ∼81% and ∼69%, respectively. Moreover, the ability to 3D print PCL-GO scaffolds with different heights enables control over cell distribution and attachment, a feature that can be also exploited for cellular confinement, i.e., for microfluidics or wound healing applications. With time, the surface wettability increases, and the scaffold can be populated by cells. Finally, the presence of GO allows for the use of infrared light for the sterilization of scaffolds and the disruption of any bacteria cell that might adhere to the more hydrophilic surface. Overall, our results showcase the potential of PCL-GO as a versatile material for medical applications.

## INTRODUCTION

The requirement for multifunctional materials for medical applications is undeniable, driven by the diverse and dynamic demands of the human body.^1^ Each specific application necessitates tailored materials that can seamlessly integrate into the complex physiological environment through specific surface and bulk functionalities. Achieving this level of versatility is of paramount importance for enhancing the safety, effectiveness, and longevity of medical devices, ultimately paving the way for innovative solutions that can address the intricate needs of *in vivo* applications. One such material that shows great promise is polycaprolactone (PCL), a biodegradable polyester that has gained attention in various biomedical fields. PCL's low toxicity, biocompatibility, and 3D printability provide an attractive choice for medical products such as absorbable sutures and wound closure devices,^2^ drug delivery systems,^3^ scaffolds for tissue engineering,^4^ such as bone,^5^ cartilage,^6^ and skin,^7^ catheters,^8^ and stents.^9,10^ Despite these advantageous properties, PCL alone lacks intrinsic antibacterial capabilities, which limit its ability to prevent infections such as surgical site infections (SSIs), that pose a substantial threat in surgical environments. Various factors significantly heighten the risk of SSIs, such as preexisting infections, extended hospital stays, suboptimal surgical procedures, prolonged surgical durations, and inadequate sterilization of surgical instruments.^11^ To address this issue, the incorporation of antibacterial materials into PCL has been explored to enhance its performance.^12^

However, biofouling, the accumulation and growth of biomacromolecules that can lead to the deterioration or impairment of implants, encompasses a wide range of lengths and time scales, making it a highly dynamic process strictly dependent on surface features.^13^ Specifically, while hydrophilicity is required to improve protein adsorption and tissue integration, hydrophobicity increases self-cleaning properties prolonging the device lifespan and reducing friction. Hydrophobic polymer surfaces can also improve long-term mechanical behavior.^14^ However, the intrinsic hydrophobicity of PCL leads to a lack of recognition sites attached by cells on the surface and limits cell spreading on scaffolds.

In summary, though the quick adsorption of proteins upon implantation serves as a foundation for microorganisms bacteria to settle and form biofilms, it also plays a crucial role in cell adhesion.^13,15^ Therefore, spatiotemporal control of biological adhesion on scaffolds is desirable to (i) initially inhibit SSI and (ii) favor cell adhesion and tissue repair on scaffolds at a later stage.

In terms of enhancing the properties of polymers, nanomaterials represent a recent solution. In this field, graphene, a two-dimensional (2D) carbon nanomaterial, and its derivatives with large surface area, high mechanical strength, excellent electrical conductivity, and inherent antibacterial effects have been largely studied as part of 3D printable composites.^16,17^ Graphene carbon atoms are spaced at a distance of 0.142 nm forming a hexagonal lattice with sp^2^ hybridization. Each atom shares three of its outer shell electrons to form covalent σ-bonds, while the fourth electron, in a p_z_ orbital, participates in the formation of a π bond. Unlike the sp^2^ orbitals, the linear combination of the p_z_ orbitals allows for a long-range delocalization of π electrons, which can move above and below the 2D graphene sheet. Such an extended delocalization of π electrons is responsible for the extraordinary electrical conductivity of graphene derivatives.^18–23^ Graphene's remarkable strength arises from its robust in-plane carbon–carbon σ-bonds. Composite materials incorporating graphene have significant advantages due to its low density (approximately 2300 kg/m^3^) and substantial Young's modulus (around 1 TPa).^24^ Graphene oxide (GO) represents an oxidized variant of graphene with the presence of oxygenated functional groups. These groups yield several consequences, including the partial disruption of the extended π-network with the creation of defects, the increase in the interlayer spacing as well as the decrease in electrical conductivity. Reduced graphene oxide (rGO), on the other hand, stands as a deoxygenated iteration of GO.^25,26^ While the reduction processes do not fully recover the pristine graphene structure, the removal of oxygen groups results in an increased C/O ratio, a tunable restoration of the sp^2^ structure, improved mechanical strength, expanded surface area, enhanced stability, hydrophobic characteristics, and an increase in electrical conductivity.^27,28^ Graphene, GO, and rGO possess distinctive bioactive properties due to their surface groups and consequently to their capacity of dissolving in apolar and polar solvents, respectively. At present, there is a consensus within the scientific community that properly designed graphene materials not only exhibit biocompatibility but also frequently outperform in creating an optimal microenvironment for cell growth and differentiation. Indeed, several graphene-based materials have been used in scaffold applications for nerve, bone, muscle regeneration, and wound healing.^29,30^

In this work, polar solvent-soluble GO, functionalized with alkylamine groups, has been used to obtain a temporally controlled bioactivity of 3D-printed PCL scaffolds. So far, the temporal control of graphene properties has been proposed in *in vitro* applications for diagnostic purpose,^31,32^ to control mechanical properties of scaffolds^33^ or to control cell or drug release^34–36^ rather than modulating cell attachment and biofouling on scaffolds. Composites of graphene derivatives and PCL have also been studied; however, frequently, GO has been reduced to rGO to allow for proper mixing with PCL^37^ since GO is poorly soluble in organic solvents compared to rGO and might result in non-uniform composites.^38,39^

In our work, the presence of alkylamine groups is used not only to enhance the dispersibility of GO in organic solvents but also to control surface hydrophobicity during contact with biological fluids. This modified graphene derivative finds applications in various fields, such as sensors and membranes, where the combination of the hydrophilic GO framework and hydrophobic alkylamine moieties can be tailored for specific interaction requirements and improved performance in diverse environments.

By incorporating 1% (w/w) GO into PCL, the PCL-GO acquires antibacterial and anti-adhesive growth properties, which are time-dependent. We demonstrate these material's features using bacteriophages, bacteria, and eukaryotic cell lines that initially cannot adhere to the printed scaffold. This phenomenon is mediated by a local increase in hydrophobicity due to the GO adsorption of solvent molecules and is fundamental to limit bacteria adhesion during surgical procedures. The phenomenon is strictly dependent also on scaffold geometry, i.e., the possibility of 3D printing in the same scaffold PCL-GO and/or PCL having different heights allows for the control of cell distribution on scaffolds. We also demonstrate that surface wettability increases after repeated contact with biological fluids, and that this consequently increases cell adhesion with time, fundamental for scaffold population and replacement *in vivo*. Due to the GO's ability to absorb infrared (IR) radiation, the scaffold can be sterilized after implantation if biofouling occurs over time. Presently, a limited number of instruments are available for regulating cell adhesion; here, we provide a simple 3D-printing method to facilitate the exploration of bottom-up tissue engineering and its spatial and temporal control.^40^

## RESULTS AND DISCUSSION

### Characterization of PCL and PCL-GO samples

PCL and PCL-GO (1 wt. %) characterization was performed with several techniques to assess the chemical, optical, morphological, and mechanical properties. The choice of concentration has been made according to literature data. Previous studies have described how a concentration of 1% of reduced GO is well tolerated by cells but, at the same time, does not cause the significant impair of mechanical properties of PCL scaffolds.^41^ This concentration is also known to induce differentiation of spindle-like MG-63 cells^42^ and has low cytotoxicity *in vitro*^43^ and *in vivo* when PCL-G has been used for the production of electric-responsive scaffolds.^44^

**FIG. 1.**
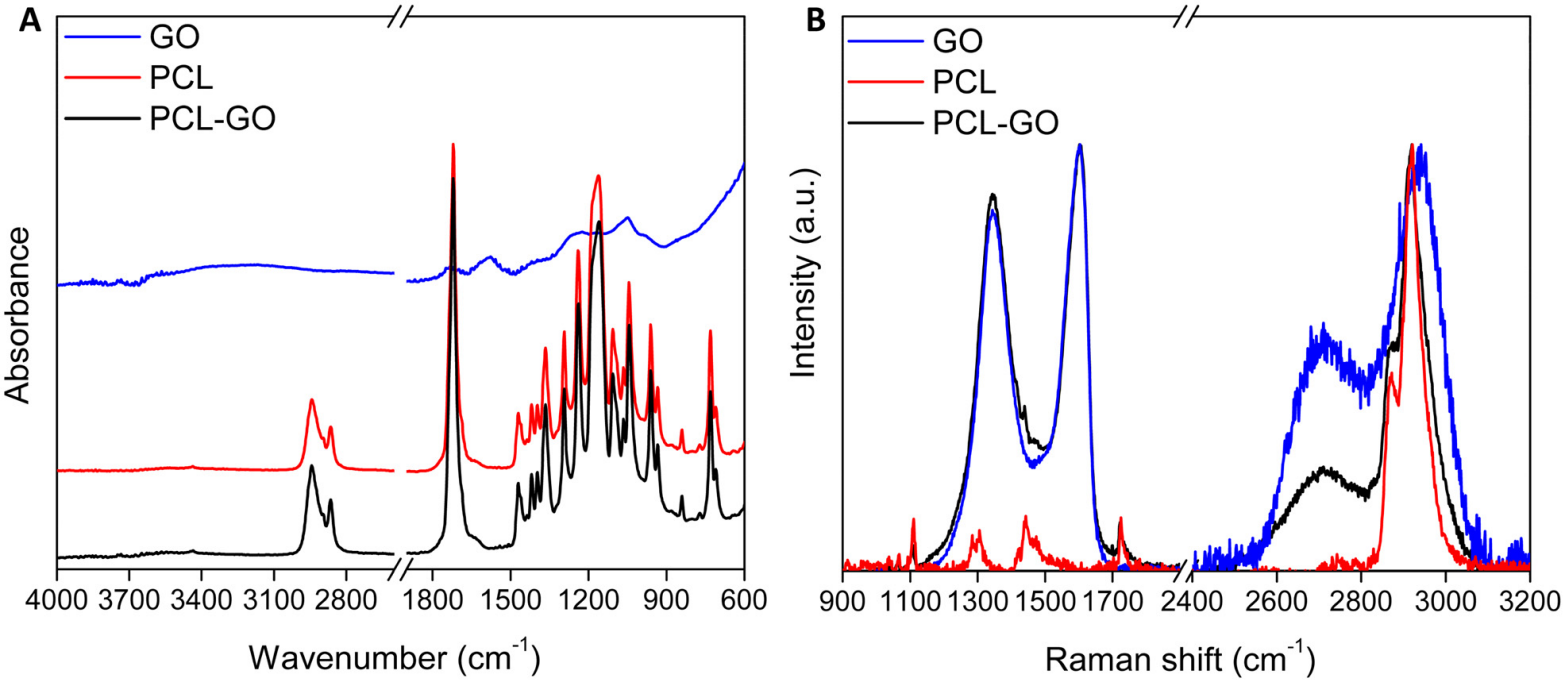
(a) FT-IR spectra of alkylamined GO (blue line), PCL (red line), and PCL-GO (black line) and (b) normalized Raman spectra of alkylamined GO (blu line), PCL (red line), and PCL-GO (black line).

In this work, polar solvent-soluble akylamined GO has been used to obtain the PCL-GO composite. The FT-IR spectrum of alkylamined GO [Fig. 1(a), black line] shows the typical band at ≈1728 cm^−1^ due to the stretching vibrations of the carbonyl groups *ν*C=O of aldehydes, ketones, and carboxyl groups, a further one at ≈1580 cm^−1^, tentatively assigned to the *ν*C=C stretching in the aromatic domains, and a last one at 1225 cm^−1^, assigned to the C–O stretching mode.^45–47^ Differently, the FT-IR spectrum of the PCL [Fig. 1(a), red line] displays vibrational bands in accordance with the literature.^48^ In particular, the most intense and narrow band localized at ≈1720 cm^−1^ and relative to the carbonyl stretching *ν*C=O almost falls in the same spectral region as that of alkylamined GO. As expected, the FT-IR spectrum of the PCL-GO composite [Fig. 1(a), blue line] prepared through a simple blending of the two species is indistinguishable from that of PCL one owing to the low amount of GO present in the composite and the absence of chemical derivatization. To gain additional information on the vibrational properties of the materials here presented, a Raman study has been performed. In particular, the Raman spectrum of alkylamined GO [Fig. 1(b), black line] displays the prominent D band localized at ≈1345 cm^−1^ due to the structural defects and G band at ≈1600 cm^−1^ related to the planar vibrations mode of the sp^2^ hybridized carbon atoms in addition to the less intense second-order 2D and D + G bands centered at ≈2706 and ≈2942 cm^−1^, respectively. The PCL spectrum [Fig. 1(b), red line] shows the typical weak peaks localized at ≈1110 cm^−1^ due to the skeletal vibrations, those within the spectral ranges between 1270 and 1320 cm^−1^ (*ω*CH_2_) and 1405–1470 cm^−1^ (*δ*CH_2_), at ≈1720 cm^−1^ (*ν*C=O), and, finally, the most intense signals relative to the symmetrical (*ν*_s_CH_2_) and asymmetrical (*ν*_as_CH_2_) stretching vibrations of the methylene groups localized at 2870 and 2920 cm^−1^, respectively.^49–52^ The aforementioned signals of the CH_2_ groups of PCL, which were also observed in the FT-IR spectra [Figs. 1(a), red and blue lines], are visible in the Raman spectrum of the PCL-GO composite [Fig. 1(b), blue line] whose intensities superimpose to the second-order peaks D + G of GO. In addition, the spectrum of PCL-GO still displays traces of the diagnostic signals of PCL, in particular those localized at ≈1110 and ≈1720 cm^−1^, overlapped to the D and G bands characteristic of GO [Fig. 1(b), blue line].^26,47,53^

**FIG. 2.**
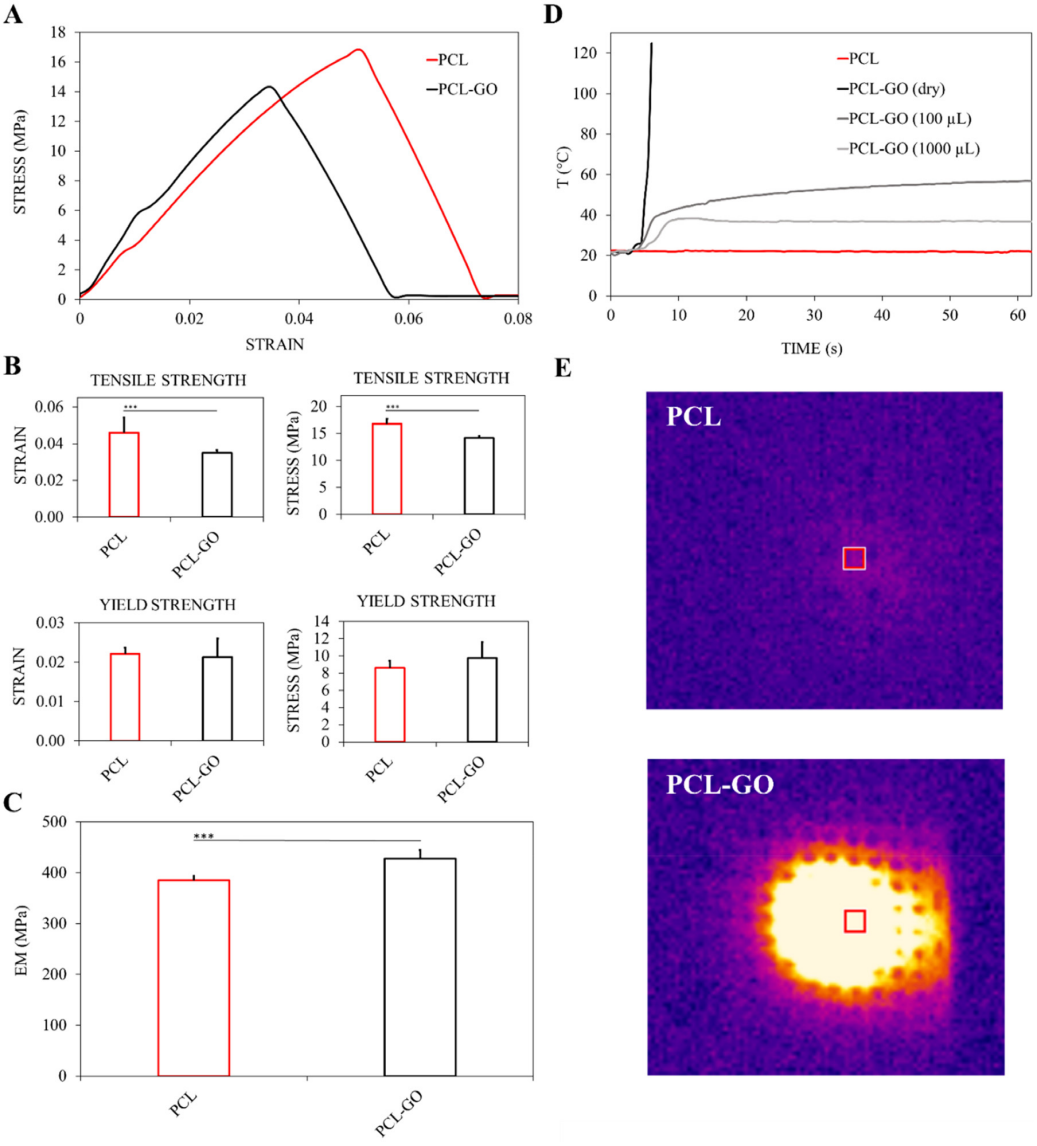
(a)–(c) Stress–strain curves of PCL and PCL-GO samples and Tensile Strength, Yield Strength, and Young's Modulus results from mechanical characterization. IR absorption (800 nm) of scaffolds (d) causes an increase in temperature for PCL-GO scaffolds in dry conditions or adding ultrapure water on the surface. A lack of IR absorption has been measured for PCL samples. (e) Representative images of PCL and PCL-GO grids exposed to NIR light obtained with thermal imaging.

The mechanical characterization of scaffolds is reported in Fig. 2. Curves obtained from tensile tests are shown in Fig. 2(a). The addition of GO at a concentration of 1% to PCL results in a ∼10% increase in Young's modulus and, consequently, a decrease in the elasticity of PCL-GO compared to PCL [Fig. 2(b)]. This results in a considerable enhancement in the stiffness of PCL-GO compared to PCL. Consequently, the addition of GO leads to a decrease in elasticity, implying that PCL-GO is less capable of returning to its original shape after deformation.

PCL-GO samples also show changes in elongation at break, a critical parameter for understanding a material's ductility and stretchability. Our data indicate a substantial reduction of approximately 23.8% in the elongation at break for PCL-GO in comparison to PCL. This reduction underscores the increased brittleness and reduced ductility of the composite material when GO is introduced [Fig. 2(b)].

We also observed a 15.6% reduction in maximum tensile stress [Fig. 2(c)] and a 12.9% increase in yield stress of PCL-GO [Fig. 2(c)], implying that it can withstand higher stresses before undergoing plastic deformation with respect to PCL.

Our data follow previous findings in the literature; indeed, Kai *et al.* used GO as an enforcing filler of PCL composites^54^ and observed a reduction in the elongation at break and a high reinforcing effect on the material, increasing the yield stress and Young's modulus with increasing volume fraction of GO. Wan and Chen investigated the mechanical behavior of PCL-GO films to exploit their potential bioactivity.^55^ They showed that adding GO at 0.3% (w/w) significantly increased tensile strength, Young's modulus, and energy at break of the PCL membrane. Akhigan *et al.* observed the same shift in the stress–strain curve with their composites of PCL and GO.^56^ In their study, they tested the mechanical changes in PCL scaffolds enriched with GO and with zinc oxide for antibacterial applications. They reported a significantly increased Young's modulus along with an improved compressive strength.

Overall, PCL-GO displays increased stiffness, decreased elasticity, reduced ductility, and lower maximum tensile stress compared to PCL.

Figure 2 shows also the response in terms of the temperature reached over time by the samples subjected to infrared laser irradiation monitored by thermal camera. Four types of samples were evaluated: PCL and PCL-GO in dry conditions, and PCL-GO coated with 100 and 1000 *μ*l of water, respectively.

While PCL samples did not exhibit a significant response to irradiation, the curves for the wet PCL-GO samples reach a plateau at 60.2 °C (PCL-GO 1000 *μ*l) and 37.2 °C (PCL-GO 100 *μ*l). In contrast, the dry PCL-GO sample rapidly heats up, reaching temperatures exceeding 120 °C within the first 10 s with consequent melting. Images taken with the thermal camera of the samples during irradiation are shown in Fig. 2(e).^57^

**FIG. 3.**
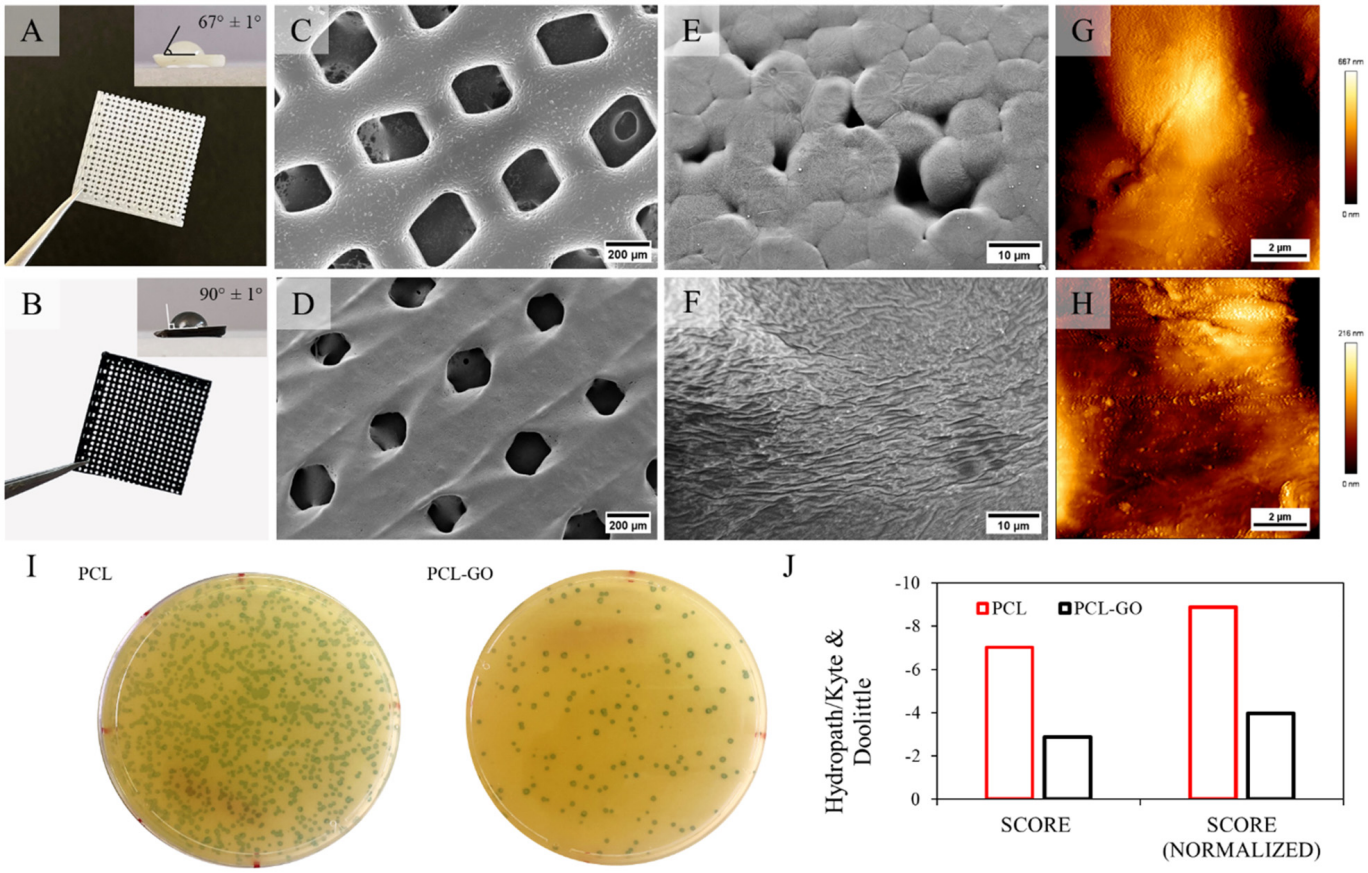
PCL (a) and PCL-GO (b) grids with respective contact angle measurements. SEM images of PCL (c) and (e) and PCL-GO (d) and (f) grid surface at 200× magnitude (c) and (d) and 5k× magnitude (e) and (f); PCL (g) and PCL-GO (h) AFM characterization of the surface. Phages recovered from each surface have been quantified using blue colonies counting on XGal (i); the average Hydropath/Kyte & Dolittle values have been calculated considering each sequence (score) or averaging per the number of phages repeated on the surface (normalized score) (j).

Figures 3(a) and 3(b) show representative PCL and PCL-GO 3D-printed grids. Contact angle measurements performed on flat samples are visible in the insets, showing the increase in hydrophobicity in PCL-GO with an increase in the contact angle.

In Figs. 3(c) and 3(d), the SEM images of grid-like structure of the PCL and PCL-GO are reported. Although the distance between the hole centers within the grid remains constant in both PCL and PCL-GO, the area of the holes shows a 49.6% reduction in the latter. The presence of GO and its heat conductive properties ensure a higher temperature uniformity during the 3D printing process, allowing heat to distribute evenly throughout the sample and producing the shown shape. This is confirmed at higher SEM magnification images [Figs. 3(e) and 3(f)], where the surface appears extremely different between PCL and PCL-GO, as confirmed by the detailed surface characterization with AFM [Figs. 3(g) and 3(h)]. Precisely, while PCL is formed by large smooth areas with discontinuities due to an uneven melting of the composite, the surface of PCL-GO has higher uniform roughness.

The roughness was assessed using AFM. The average roughness obtained for PCL on 10 × 10 *μ*m^2^ area images is 35 ± 4 nm, whereas it is 100 ± 20 nm for PCL-GO. The results obtained confirm the observations made on SEM images and show an increase in the average roughness for PCL-GO (+286%) compared to PCL. We hypothesize that the different surface morphology is also explained by the GO-modified heat transfer during the 3D printing.

Furthermore, we screened an M13-based phage library to select the most frequent peptides that specifically bind to the scaffolds and to obtain insights about the hydrophobic characteristics of the surface. In Fig. 3(i), we show the X-Gal Agar plates obtained after infection of *E. coli* with phages eluted after three rounds of biopanning. It is visible how the number of colonies is reduced on PCL-GO (10^4^ plaques/ml compared to 10^5^ plaques/ml for PCL), indicating a smaller amount of phages attached to the surface. Sequence analysis of the peptides enriched during biopanning showed that the aminoacidic stretch of PCL has higher hydrophilicity compared to PCL-GO whose hydrophobicity reduces phage binding [Fig. 3(j)]. The hydrophobicity was calculated as the total average of the amino acids and from the weighted total score for the repeated peptide sequences from the phage recovered. All the indexes confirm a better hydrophilicity for PCL.

### Temporal control of PCL-GO scaffolds interaction with eukaryotic cells

**FIG. 4.**
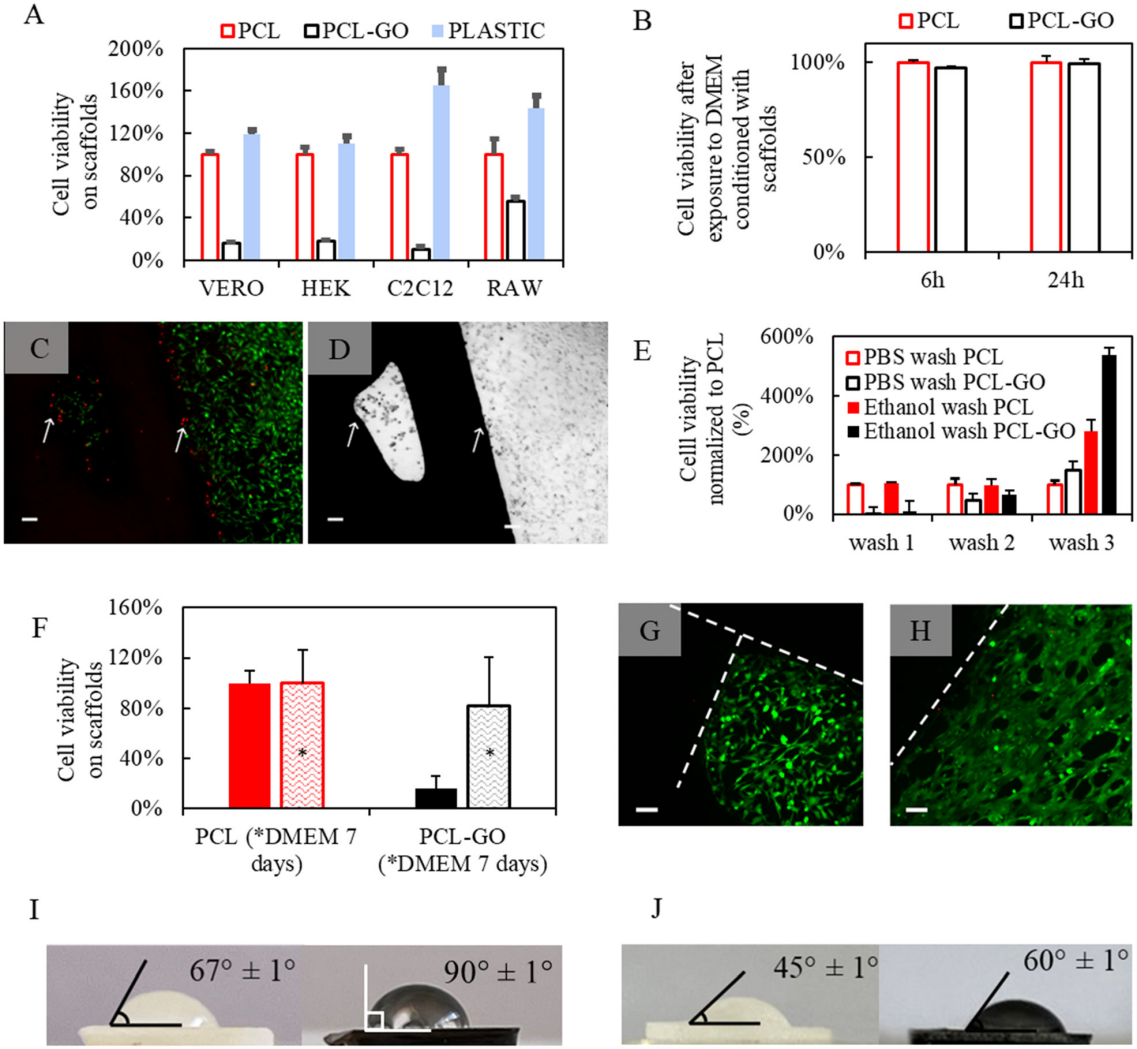
(a) Viability of different cell lines grown on scaffolds or plastic wells evaluated by luminescence assay. (b) Evaluation of toxicity of DMEM exposed to scaffolds for 7 days and used to treat VERO cells seeded on 96 wells. Toxicity on VERO cells has been evaluated after 6 or 24 h of treatment. (c) Fluorescence imaging of VERO cells surrounding grids of PCL-GO. Cells have been labeled with calcein (green) to evaluate viable cells and propidium iodide (red) to evaluate dead cells. Arrows indicate boundaries between the scaffold and well. The brightfield image of the same sample is shown in (d). (e) Evaluation of VERO cell viability on scaffolds washed with different protocols (PBS or PBS+ethanol). (f) Cell viability on the scaffold after washing with DMEM (indicated with asterisks) or without DMEM washings. (g) and (h) Representative fluorescence images of cells surrounding grids (dashed lines) after DMEM washings, no red dead cells are visible. Scale bar is 100 μm. Contact angles after one wash in ethanol (i) and after three washes in ethanol (j) show an increased hydrophilicity in the latter case.

To evaluate the biocompatibility, VERO, HEK, C2C12, and RAW cell lines were plated on PCL and PCL-GO scaffolds, and cellular viability was evaluated after 72 h by bioluminescence after moving the scaffolds in a new well to quantify the signal from cells directly attached to the surface [Fig. 4(a)]. It was observed that the cellular adhesion on PCL-GO was significantly lower compared to PCL samples. As a control, in Fig. 4(a), the value of cell viability on plastic wells is reported, as expected adhesion on plastic is systematically higher compared to PCL.

PCL is, indeed, relatively hydrophobic, and we have shown how this hydrophobicity increases for PCL-GO samples (Fig. 3). To achieve good cell adhesion, it has been noted that the ideal range for water contact angle values falls between 45 and 70 °C. This is because very high contact angles and low surface energy result in diminished cell-conductive behavior and protein denaturation.^58^ Therefore, we hypothesize that the cell adhesion on PCL-GO is reduced due to the hydrophobicity that limits cell contacts with the surface.

However, we wanted to verify toxicity related to the incorporation of GO solubilized with a polar solvent into PCL material. As shown in Fig. 4(b), the toxicity toward VERO cells of the DMEM conditioned with the PCL-GO scaffolds for 7 days is not significantly higher compared to cells treated with DMEM conditioned with PCL scaffold. In other words, even after 7 days of scaffold submersion in DMEM, there is no significant release of toxic molecules in the medium.

However, in Fig. 4(c), from fluorescence images of cells labeled with calcein and propidium iodide, a local cytotoxic effect of PCL-GO scaffolds is visible: cells in red, i.e., dead cells, are distributed along scaffolds borders as shown also in Fig. 4(d) by brightfield image of the sample. The red signal from dead cells disappears with increased distance. Consequently, the scaffold inhibits cell adhesion and has a local inhibition of growth but is not toxic toward the cells grown in the same petri since conditioned DMEM toxicity is negligible [Fig. 4(b)].

The advantages of decreased cell adhesion in PCL-GO include minimizing the risk of biofouling, a common issue in biomedical applications, especially in the surgical environment, where bacterial contamination is facilitated. However, it is important to control long-term cell adhesion to ensure scaffold biodegradability and population over time, paramount in promoting successful tissue regeneration and minimizing the risk of necrotic infections. To understand the process and to verify the long-term effect of local cytotoxicity, we repeatedly washed the scaffolds with different protocols.

In Fig. 4(e), a comparison of washing with PBS or PBS+ethanol is shown for PCL and PCL-GO. We observe, with the increase in the number of washes, a notable increase in cell attachment on the scaffold, especially with washes with PBS+ethanol. The use of ethanol, in which the dichloromethane (DCM) is soluble, allows for a quicker removal of DCM residues. After three washes, the cell adhesion is markedly increased, and the PCL-GO can increase cell attachment five times more than PCL, as reported in the literature for other graphene-enriched materials.^59^ Therefore, the residuals of polar solvents persist on GO due to its ability to act as a surfactant.

*In vivo*, the removal of DCM and the change of the surface will likely occur in an environment rich in salts, plasma proteins, and nutrients. To simulate this, we repeatedly washed the scaffold with DMEM growth medium. After 1 week of washing, the bioconductivity of PCL-GO scaffolds reaches that of PCL [Fig. 4(f)].

Accordingly, red dead cells are not visible around the grid in fluorescence images of VERO cells grown on DMEM washed scaffolds [Figs. 4(g) and 4(h)].

This phenomenon can be explained by a change in the hydrophilicity after repeated washings of scaffolds as shown in Figs. 4(i) and 4(j) with contact angle measurement. We therefore hypothesize, after washes, a combined effect of (i) a reduced amount of solvent, (ii) an increase in the hydrophilicity, and (iii) the rough morphology of PCL-GO observed with surface characterization in Fig. 2. We point out that this result is significant for the 3D printing of graphene and GO and more, in general, for the 3D printing of DCM-solubilized PCL scaffolds that also gain a certain degree of hydrophilicity after washings [Fig. 4(e)].

We then 3D-printed scaffolds directly into petri dishes using different scaffold heights.

**FIG. 5.**
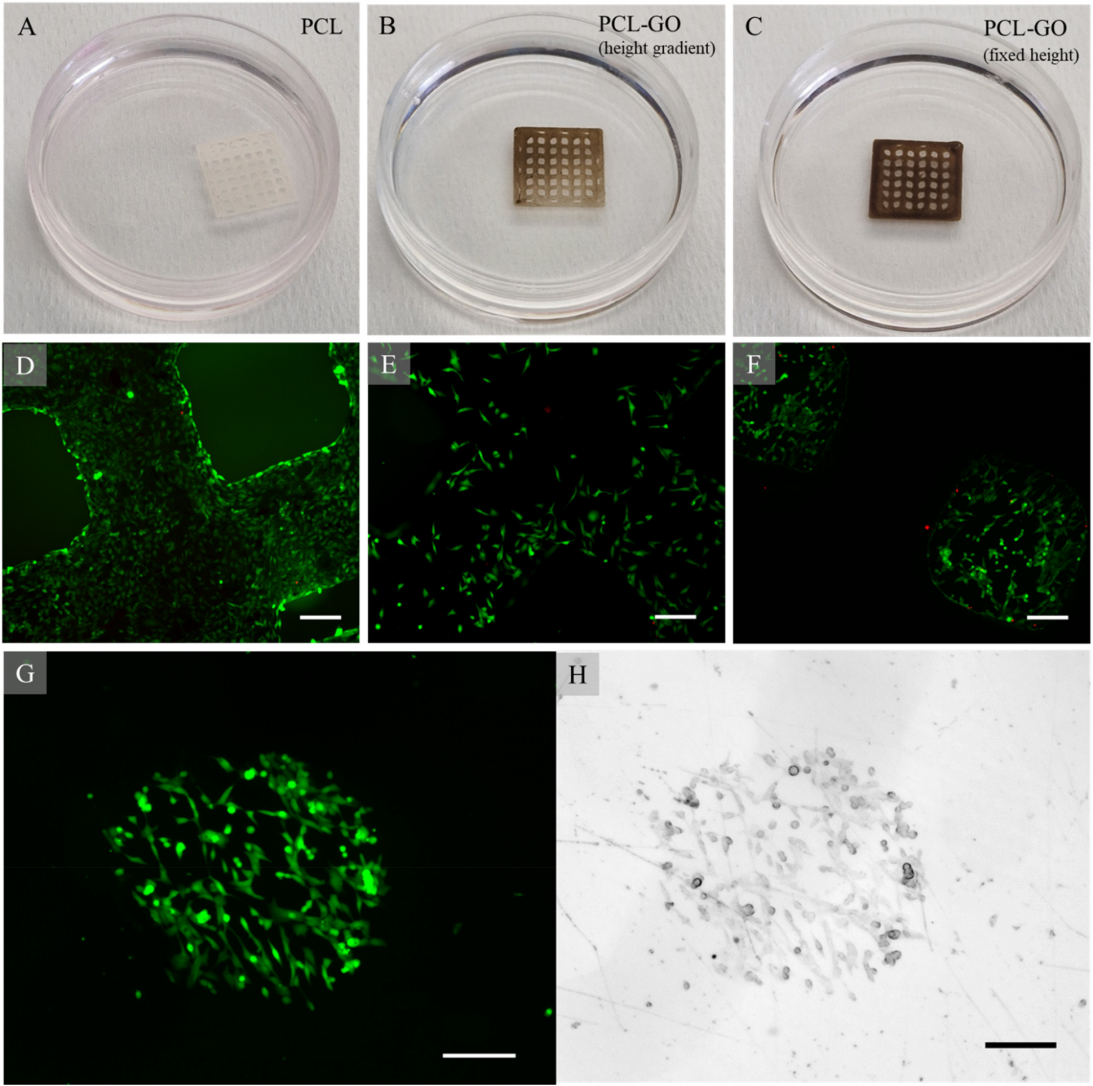
Images of PCL grids (a) or PCL-GO grids (b) and (c) printed at different heights. Fluorescence images of grids areas with 50 (d), 100 (e), or 200 *μ*m height (f). Fluorescence (g) and brightfield (h) representative images of cells confined in the area defined by the PCL-GO grid. The scale bar is 100 *μ*m.

We observed a sudden detachment from the surface for PCL scaffolds, probably since the PCL-GO is more hydrophobic or due to the more homogeneous nature of PCL-GO composite that improves scaffold adhesion to plastic [Fig. 5(a)].

For PCL-GO, we prepared a scaffold having different heights: a gradient from 50 to 100 *μ*m [Fig. 5(b)] or constant height 200 *μ*m [Fig. 5(c)] and washed surfaces with DMEM multiple times. Interestingly, we observed a cell distribution according to height: while cells attached easily to flat scaffold surfaces [Fig. 5(d)], in other cases, we observed that the higher the height, the lower the cellular adhesion [Figs. 5(e) and 5(f)], with a complete evading of the grid area for 200 *μ*m high scaffolds [Fig. 5(f)]. We hypothesize, due to the absence of dead cells in Figs. 5(d) and 5(e), an effect of DCM removal proportional to the area exposed to DMEM washing. This would allow *in vivo* to foresee the cellular distribution according to the height of the surface of the scaffold coatings/device thickness, allowing to increase the cellular adhesion in precise scaffold areas.

This phenomenon can also be exploited to 3D print grids directly into petri dishes for cell confinement in experiments, like wound healing or microfluidics assays. The grid can then be removed, and the islet of cells can be obtained on petri surfaces, as shown as proof of concept in Figs. 5(g) and 5(h).

### Limiting infections on implants: Antibacterial effects of PCL-GO scaffolds

Surgical infections can arise through two primary routes: contiguously and hematogenously. Contiguous contamination occurs during the implantation process itself, where microorganisms from the surrounding environment may inadvertently come into contact with the scaffold. Hematogenous spread, on the other hand, involves the introduction of infectious agents via the bloodstream. While the body's natural defenses typically prevent such systemic infections, certain factors, such as compromised immune function or preexisting infections, can increase the risk. Therefore, meticulous attention to both aseptic techniques during surgery and the design of implantable materials that discourage microbial adhesion is crucial in minimizing the potential for surgical site infections.

**FIG. 6.**
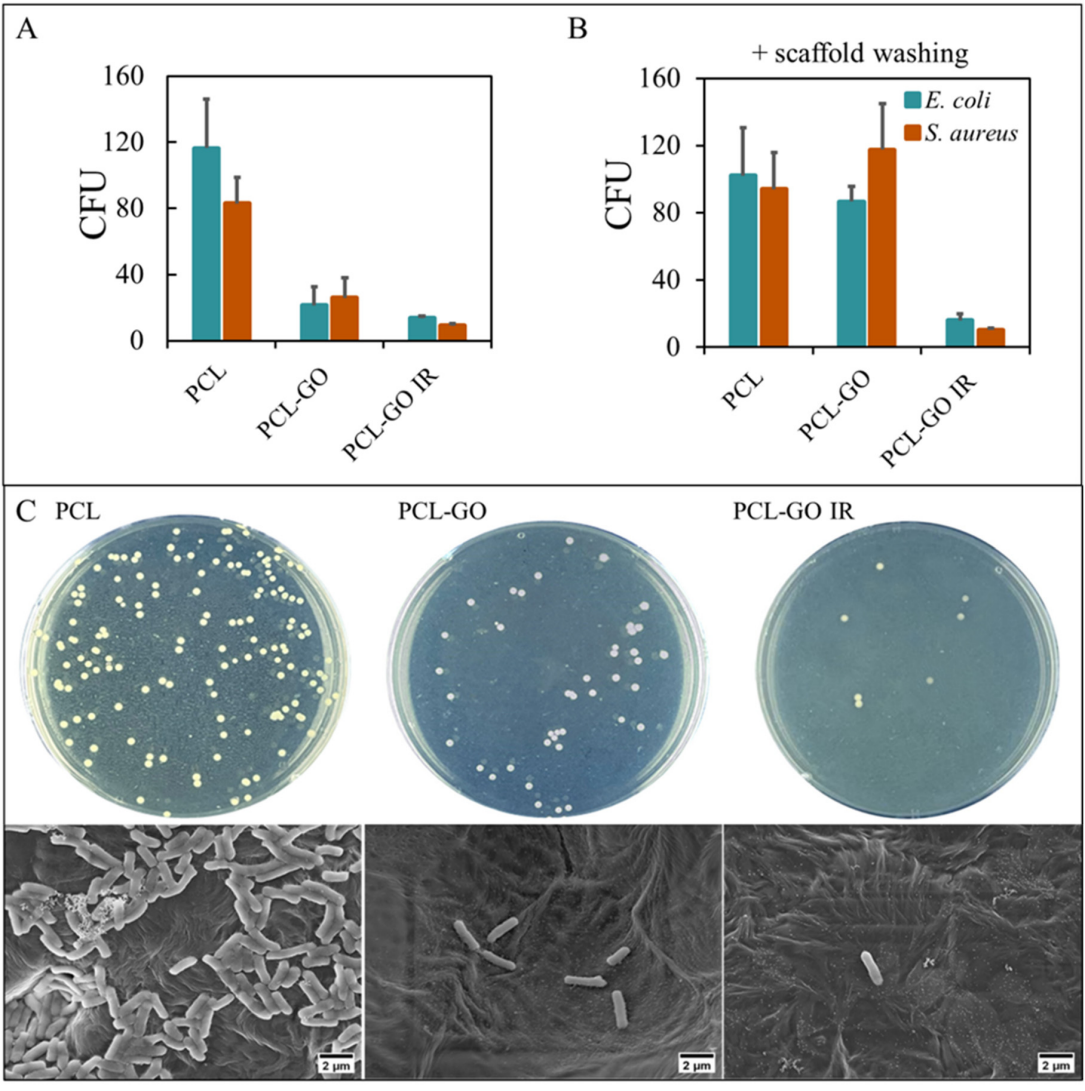
Antibacterial effects of PCL and PCL-GO scaffolds, with or without IR irradiation, on *E. coli* or *S. aureus* cells seeded on scaffolds (a). After scaffold washing, the CFUs have been measured and reported in (b), the CFU/ml is CFU*10^5^ according to the dilution used for plating. Representative images of CFU plates and SEM imaging for *E. coli* (c).

To test the antibacterial properties of PCL and PCL-GO scaffolds, *E. coli* or *S. aureus* cells were deposited on scaffolds and let interact in a controlled environment as described in the Methods section. The results in terms of CFU collected from surfaces are shown in Fig. 6(a).

On fresh surfaces, on PCL-GO, there is a reduction of ∼81% concerning the number of *E. coli* cells and a ∼69% reduction of *S. aureus* cell number compared to PCL, demonstrating PCL-GO as an excellent candidate for infection control during the initial implantation, which is fundamental for infections derived from surgical environment. IR treatment of PCL-GO surfaces does not significantly improve the antibacterial efficacy of PCL-GO at this stage.

The long-term efficacy of surfaces has been tested after repeated washing, to assess whether the antibacterial effect is preserved over a prolonged time *in vivo*. As reported for eukaryotic cells, the washing of the surfaces induces modifications that improve hydrophilicity and consequently bacterial adhesion [Fig. 6(b)]. However, thanks to the IR adsorptive properties of PCL-GO, the antibacterial effect is restored after 30 s of treatment both for *E. coli* and *S. aureus*. Representative images of CFU plates and SEM images of *E. coli* grown on surfaces are shown in Fig. 6(c).

The addition of GO on surfaces has been often reported to induce antibacterial properties: GO is, indeed, known to affect the cell membrane and cell wall of microorganisms by producing ROS and through physical demolition and chemical oxidation, resulting in microbial death.^60^ This is, however, a phenomenon well comprised in soluble GO experiments.^61,62^ On scaffolds, the antibacterial efficacy of GO is more likely proportional to the amount of GO surface available to interact with bacterial cells.^63,64^ In this case, we hypothesize that as for cells, the DCM residuals are responsible for the antibacterial effects that are lost after repeated washes. Even if the antibacterial efficacy would gradually be lost *in vivo*, the IR absorptive properties of GO can be used to reduce any long-term infection that might occur after implantation as demonstrated in Fig. 6(c).

## CONCLUSIONS

The demand for versatile materials in medicine is evident across a wide spectrum of applications, ranging from supporting structures like bones to delicate interactions with soft tissues. A significant challenge in medical settings is the occurrence of biofouling, which involves the unwanted buildup and proliferation of microorganisms on implanted materials. In addition to the need to counter infections, it is imperative to integrate scaffolds *in vivo* over time. This integration is essential for enabling tissue regeneration and ensuring the proper functioning of biomedical implants.

In this work, we demonstrate that the addition of a small percentage of GO to PCL allows to 3D print scaffolds with multiple functionalities. GO improves the mechanical performance of PCL and infers IR absorption properties.^17,65^ The added GO dramatically changes the surface features due to the heat conductivity improvement during 3D printing and to the retainment of small amounts of DCM that increase hydrophobicity and toxicity of the surface. This is advantageous to prevent fouling from bacteria in the surgical site. It should be pointed out that this toxicity is spatially limited to the few micrometers around the scaffold surface since cells seeded in the proximity of the scaffold grow undisturbedly. When the surface is put in contact with fluids rich in proteins and nutrients, a phenomenon that occurs in all medical devices intended for long-term use *in vivo*, the toxicity is progressively loss according to the thickness of the scaffold: this allows us to foresee cell attachment behavior and ultimately tissue integration over time. In turn, also antibacterial effects of PCL-GO scaffolds, which are initially very high, are limited when the surface is put in contact with the growth medium for a prolonged period. However, the IR absorption by the scaffold can be used to locally increase temperature and destroy bacterial cells by hyperthermia. This method can ensure bacterial elimination in cases of a secondary infection via hematogenous spreading, as an example.

We highlight that the low cell attachment feature of PCL-GO can be also exploited to create confined cell areas for experiments like wound healing or invasion assays, and co-cultures to replicate tissue structures by modulating scaffold height and/or composition.

The increase in hydrophilicity obtained for PCL and PCL-GO after repeated washings represents a time-controlled low-cost simple strategy compared to protein coating, cold plasma treatment, and chemical etching. Looking ahead, this research paves the way for the development of advanced biomaterials with diverse applications in tissue engineering and medical device design.

## METHODS

### Materials

The materials used were Alkylamined Graphene Oxide (GO S-921556, Sigma-Aldrich), dichloromethane (DCM, Carlo Erba), ethanol (Carlo Erba), polycaprolactone (PCL, 43–50 kDa, hydroxyl end group. mp 55–65° C, Polysciences, Inc), African green monkey kidney epithelial cells (VERO) (ATCC CCL-81), Dulbecco's Modified Eagle's Medium (DMEM) (Sigma-Aldrich, St. Louis, MO, USA), fetal bovine serum (FBS) EuroClone, streptomycin–penicillin (EuroClone, Milan, Italy), and Murine myoblast C2C12 cells American Type Culture Collection (ATCC). Differentiation medium (DM), made of DMEM, 2% Horse Serum (HS), 100 U/ml penicillin, and 100 *μ*g/ml streptomycin (EuroClone, Milan, Italy), HEK-Dual™ Null (NF/IL8) cells (Invivogen), RAW 264.7 murine macrophage cell line (ATCC^®^ CRL1469™), CellTiter-Glo^®^ Luminescent Cell Viability Assay (Promega, Madison, WI, USA), *E. coli* (ATCC 25922), *S. aureus* (ATCC 29213), LB Broth medium (Sigma-Aldrich), Ph.D.-12 Phage display library kit (New England Biolabs), Propidium Iodide (Sigma-Aldrich), Calcein, AM, cell-permeant dye (Invitrogen™).

### 3D printing

3D printing of scaffolds was performed with a BIO X 3D bioprinter (Cellink). PCL (900 mg) and GO (9 mg) were dissolved in 20 ml of DCM in glass bottles under stirring for 2 h. Then, solutions were mixed under stirring for 1 h, and a GO 1% w/w concentration was obtained after solvent evaporation. The mixture (PCL-GO) was air-dried in large Petri dishes, the produced film was cut into small pieces, and then, it was transferred to a thermoplastic printhead (Cellink, heating capacity of up to 250 °C). The structure of scaffolds was designed using modeled 3D computer graphics and computer-aided design (CAD) software Rhinoceros software (Robert McNeel & Associates). The extrusion-based printing was done using a printhead temperature of 65 °C and a printbed temperature of 25 °C. The extrusion pressure was set at 40 kPa, with a pre-flow of 20 ms and a speed of 22 mm/s, and the nozzle diameter was 200 *μ*m.

### FT-IR and Raman spectroscopy

The chemical analysis of PCL and PCL-GO was carried out using attenuated total reflectance-Fourier transform infrared spectroscopy (ATR-FTIR) Bruker ALPHA II compact FTIR Spectrometer, equipped with an attenuated total reflection module (Eco-ATR). The material under investigation was directly laid upon the ATR crystal, and the spectra were recorded in the wave number range of 4000–550 cm^−1^, with a resolution of 2 nm. Raman spectra were run at room temperature in backscattering geometry with an inVia Renishaw micro-Raman spectrometer equipped with an air-cooled CCD detector and super-Notch filters. An Ar^+^ ion laser (λ_laser_ = 514 nm) was used, coupled to a Leica DLML microscope with a 20× objective. The resolution was 2 cm^−1^, and spectra were calibrated using the 520.5 cm^−1 ^line of a silicon wafer. Raman spectra were acquired in several different spots on the surface of the samples. For GO, PCL, and PCL-GO composite, each spectrum was acquired with 1% of power, 10 s of spectral acquisition, and 20 scans.

### Mechanical properties

Mechanical testing of samples was performed to retrieve tensile strength (TS), elongation at break (EB), and elastic modulus (EM) using 3D printed dog-bone-shaped specimens and a mechanical analyzer (UniVert CellScale system, Canada). The grip separation was 20 mm, and the speed rate was1 mm/s until breaking. At least three samples for each condition were used.^66^

### IR photothermal properties

To assess the photothermal properties of 3D printed materials, samples were irradiated under an 808 nm (diode Laser Ever, China) for different time spans at a power density of 1.6 W/cm^2^. A thermal imaging camera (Xi400, Optris) was used to record the sample temperature. All tests were performed in triplicate.

### Wettability

Contact angle measurements were performed on each material surface using the drop shape analysis method^66^ using 10 *μ*l of de-ionized water and the instrument described in the literature.^67^

### Morphological characterization of samples

To perform imaging, samples were first cleaned to remove any contaminants or debris using ethanol and then rinsed with distilled water to remove any residual ethanol. The samples were deposited on sterile mica slides and air-dried overnight.

Atomic force microscopy (AFM) was performed with a NanoWizard II (JPK Instruments AG, Berlin, Germany) in contact mode. The images were acquired using silicon cantilevers with high aspect-ratio conical silicon tips (CSC37 Mikro-Masch, Tallinn, Estonia) characterized by an end radius of about 10 nm, a half conical angle of 20°, and a spring constant of 0.6 N/m. Scan areas of 10 × 10 *μ*m^2^ were imaged.

The surface roughness of all samples was evaluated using the software JPK SPM Data Processing. Three areas were imaged with AFM for each sample, and the roughness was measured in terms of both the arithmetical mean deviation of the assessed profile (Ra) and of root mean squared (Rq).

Scanning electron microscopy (SEM) was performed to evaluate 3D-printed scaffold morphology. All the samples were sputter coated with a layer of 100 nm of gold. Images have been acquired with SEM Supra 25 (Zeiss, Germany) at several magnifications (scale bars are reported on each image). Images were analyzed using FIJI software (National Institutes of Health, Bethesda, MD, USA). For imaging of bacteria cells, samples were fixed in glutaraldehyde (2.5%), dehydrated in ethanol series, and dried and sputter coated with 150 nm of gold.

### Cell cultures, cell adhesion, and toxicity evaluation

VERO, C2C12, RAW 264.7, and HEK cells were cultured in DMEM supplemented with 10% FBS, 2% streptomycin/penicillin antibiotics, and in a humidified atmosphere (5% CO_2_, 37 °C). To investigate cell attachment on PCL and PCL-GO, VERO, C2C12, HEK, and RAW cells were cultured on PCL and PCL-GO supports, and viability was assessed after 72 h by using CellTiter-Glo^®^ Luminescent Cell Viability Assay according to manufacturer's instructions. Plastic was used as control. To measure the cell viability, CellTiter-Glo was added to each well with a volume equal to culture medium and shaken for 2 min in an orbital shaker to induce cell lysis. Plates were incubated at room temperature for 10 min before recording luminescence using a Cytation 3 Cell Imaging Multi-Mode Reader (Cytation 3, Biotek, USA).

### Antibacterial effects

Samples were tested for their antibacterial performance. *E. coli* ATCC 25922 or *S. aureus* ATCC 29213 adhesion on surfaces was quantified using the colony counting method as previously reported.^68^ Bacteria were inoculated in a Lennox LB Broth at 37 °C overnight. Afterward, 250 *μ*l of cell suspension was subcultured in 250 ml of LB, and then cells were harvested at the exponential growth phase and diluted in PBS. PCL and PCL-GO samples were incubated with 50 *μ*l of bacteria suspension diluted in PBS at a concentration of 10^5^ CFU/ml and incubated for 3 h. At the end of the incubation, the samples were washed and vortexed in PBS to recover cells from the surface. The resulting solution was cultured on LB Agar plates and incubated at 37 °C overnight. After incubation, the CFUs were quantified. For IR treatment, scaffolds were exposed to IR light at a power density of 1.6 W/cm^2^ for 30 s. Each experiment was repeated in triplicate.

### Phage adhesion and phage display

A library of random peptides, 12 amino acids long displayed on the minor coat protein, gene III, of the bacteriophage M13 was used for phages experiments (Ph.D.-12 Phage display library kit). *E. coli* host strain K12ER2738 was used for plating and propagation into LB/tetracycline medium plates, using overnight incubation at 37 °C. A polyethylene 96-wells plate was used for the panning procedure with different scaffolds PCL or PCL-GO surfaces. Each well was filled with 300 *μ*l blocking buffer (0.1 M NaHCO_3_ pH 8.6) and incubated for 1 h at 4 °C; then, the wells were rapidly washed six times with 300 *μ*l TBST (tris buffered saline-tween). 100 *μ*l of the phage library solution was pipetted into the coated wells and gently rocked for 1 h at room temperature. Unbounded phages were removed by washing with 300 *μ*l TBST ten times. To elute the bounded phages, 100 *μ*l of 0.2 M glycline-HCl (pH 2.2) and 1 mg/ml BSA were added to each well and incubated for 8 min upon gently rocking; then, the pH of the eluate was neutralized with 15 *μ*l 1M Tris-HCl (pH 9.1). Subsequently, the eluted phage solution was tittered using 200 *μ*l *E. coli* strain ER2738, grown in LB medium at 37 °C. After that, the infected cells were transferred to culture tubes containing melted top agar, vortexed, and poured on LB/IPTG/Xgal plates for incubation at 37 °C. After the third round of biopanning, predominant scaffold-binding selective phages were isolated and sequenced.^69^

## Data Availability

The data that support the findings of this study are available from the corresponding authors upon reasonable request.

## References

[1] J. L. Gerardo‐Nava , J. Jansen , D. Günther , L. Klasen , A. L. Thiebes , B. Niessing *et al.*, “ Transformative materials to create 3D functional human tissue models in vitro in a reproducible manner,” Adv. Healthcare Mater. 12, 2301030 (2023). 10.1002/adhm.202301030 PMC11468549 37311209

[2] X. Deng , M. Gould , and M. A. Ali , “ Fabrication and characterisation of melt-extruded chitosan/keratin/PCL/PEG drug-eluting sutures designed for wound healing,” Mater. Sci. Eng. C 120, 111696 (2021). 10.1016/j.msec.2020.111696 33545855

[3] P. Grossen , D. Witzigmann , S. Sieber , and J. Huwyler , “ PEG-PCL-based nanomedicines: A biodegradable drug delivery system and its application,” J. Control Release 260, 46–60 (2017). 10.1016/j.jconrel.2017.05.028 28536049

[4] N. Siddiqui , S. Asawa , B. Birru , R. Baadhe , and S. Rao , “ PCL-based composite scaffold matrices for tissue engineering applications,” Mol. Biotechnol. 60, 506–532 (2018). 10.1007/s12033-018-0084-5 29761314

[5] S.-R. Son , N.-T. B. Linh , H.-M. Yang , and B.-T. Lee , “ *In vitro* and *in vivo* evaluation of electrospun PCL/PMMA fibrous scaffolds for bone regeneration,” Sci. Technol. Adv. Mater. 14, 015009 (2013). 10.1088/1468-6996/14/1/015009 27877567 PMC5090585

[6] Z. Yin , D. Li , Y. Liu , S. Feng , L. Yao , X. Liang *et al.*, “ Regeneration of elastic cartilage with accurate human-ear shape based on PCL strengthened biodegradable scaffold and expanded microtia chondrocytes,” Appl. Mater. Today 20, 100724 (2020). 10.1016/j.apmt.2020.100724

[7] A. Ghaee , S. Bagheri-Khoulenjani , H. A. Afshar , and H. Bogheiri , “ Biomimetic nanocomposite scaffolds based on surface modified PCL-nanofibers containing curcumin embedded in chitosan/gelatin for skin regeneration,” Composites, Part B 177, 107339 (2019). 10.1016/j.compositesb.2019.107339

[8] E. M. J. Lin , C. L. Lay , G. S. Subramanian , W. S. Tan , S. S. J. Leong , L. C. H. Moh *et al.*, “ Control release coating for urinary catheters with enhanced released profile for sustained antimicrobial protection,” ACS Appl. Mater. Interfaces 13, 59263–59274 (2021). 10.1021/acsami.1c17697 34846837

[9] D. S. Chan , N. Fnais , I. Ibrahim , S. J. Daniel , and J. Manoukian , “ Exploring polycaprolactone in tracheal surgery: A scoping review of in-vivo studies,” Int. J. Pediatr. Otorhinolaryngol. 123, 38–42 (2019). 10.1016/j.ijporl.2019.04.039 31059931

[10] A. J. Guerra , P. Cano , M. Rabionet , T. Puig , and J. Ciurana , “ 3D-printed PCL/PLA composite stents: Towards a new solution to cardiovascular problems,” Materials 11, 1679 (2018). 10.3390/ma11091679 30208592 PMC6164695

[11] K. M. Ali and B. M. A. Al-Jaff , “ Source and antibiotic susceptibility of gram-negative bacteria causing superficial incisional surgical site infections,” Int. J. Surg. Open 30, 100318 (2021). 10.1016/j.ijso.2021.01.007

[12] L. Liu , Y. Zhang , C. Li , J. Cao , E. He , X. Wu *et al.*, “ Facile preparation PCL/modified nano ZnO organic-inorganic composite and its application in antibacterial materials,” J. Polym. Res. 27, 78 (2020). 10.1007/s10965-020-02046-z

[13] C. M. Magin , S. P. Cooper , and A. B. Brennan , “ Non-toxic antifouling strategies,” Mater. Today 13, 36–44 (2010). 10.1016/S1369-7021(10)70058-4

[14] M. M. Hossain and V. R. Lokasani , “ Improving the hydrophobicity of polymers through surface texturing,” in Conference Proceedings Society of Plastics Engineers, 2021. PMC9075775 35529586

[15] V. Palmieri , M. De Spirito , and M. Papi , “ Nanofeatures of orthopedic implant surfaces,” Future Med. 16, 1733–1736 (2021). 10.2217/nnm-2021-0118 34196227

[16] G. Perini , A. Rosenkranz , G. Friggeri , D. Zambrano , E. Rosa , A. Augello *et al.*, “ Advanced usage of Ti_3_C_2_T_x_ MXenes for photothermal therapy on different 3D breast cancer models,” Biomed. Pharmacother. 153, 113496 (2022). 10.1016/j.biopha.2022.113496 36076510

[17] V. Palmieri , F. Sciandra , M. Bozzi , M. De Spirito , and M. Papi , “ 3D graphene scaffolds for skeletal muscle regeneration: Future perspectives,” Front. Bioeng. Biotechnol. 8, 383 (2020). 10.3389/fbioe.2020.00383 32432094 PMC7214535

[18] A. K. Geim and K. S. Novoselov , “ The rise of graphene,” Nat. Mater. 6, 183–191 (2007). 10.1038/nmat1849 17330084

[19] Y. Zhang , Y.-W. Tan , H. L. Stormer , and P. Kim , “ Experimental observation of the quantum Hall effect and Berry's phase in graphene,” Nature 438, 201–204 (2005). 10.1038/nature04235 16281031

[20] K. S. Novoselov , A. K. Geim , S. V. Morozov , D. Jiang , M. I. Katsnelson , I. V. Grigorieva *et al.*, “ Two-dimensional gas of massless Dirac fermions in graphene,” Nature 438, 197–200 (2005). 10.1038/nature04233 16281030

[21] J.-H. Chen , C. Jang , S. Xiao , M. Ishigami , and M. S. Fuhrer , “ Intrinsic and extrinsic performance limits of graphene devices on SiO_2_,” Nat. Nanotechnol. 3, 206–209 (2008). 10.1038/nnano.2008.58 18654504

[22] K.-J. Tielrooij , J. C. W. Song , S. A. Jensen , A. Centeno , A. Pesquera , A. Zurutuza Elorza *et al.*, “ Photoexcitation cascade and multiple hot-carrier generation in graphene,” Nat. Phys. 9, 248–252 (2013). 10.1038/nphys2564

[23] A. H. C. Neto , F. Guinea , N. M. R. Peres , K. S. Novoselov , and A. K. Geim , “ The electronic properties of graphene,” Rev. Mod. Phys. 81, 109 (2009). 10.1103/RevModPhys.81.109

[24] C. Lee , X. Wei , J. W. Kysar , and J. Hone , “ Measurement of the elastic properties and intrinsic strength of monolayer graphene,” Science 321, 385–388 (2008). 10.1126/science.1157996 18635798

[25] V. Palmieri , E. A. Dalchiele , G. Perini , A. Motta , M. De Spirito , R. Zanoni *et al.*, “ Biocompatible *N*-acetyl cysteine reduces graphene oxide and persists at the surface as a green radical scavenger,” Chem. Commun. 55, 4186 (2019). 10.1039/C9CC00429G 30892320

[26] F. Amato , G. Perini , G. Friggeri , A. Augello , A. Motta , L. Giaccari *et al.*, “ Unlocking the stability of reduced graphene oxide nanosheets in biological media via use of sodium ascorbate,” Adv. Mater. Interfaces 10, 2300105 (2023). 10.1002/admi.202300105

[27] A. T. Smith , A. M. LaChance , S. Zeng , B. Liu , and L. Sun , “ Synthesis, properties, and applications of graphene oxide/reduced graphene oxide and their nanocomposites,” Nano Mater. Sci. 1, 31–47 (2019). 10.1016/j.nanoms.2019.02.004

[28] B. Lesiak , G. Trykowski , J. Tóth , S. Biniak , L. Kövér , N. Rangam *et al.*, “ Chemical and structural properties of reduced graphene oxide—Dependence on the reducing agent,” J. Mater. Sci. 56, 3738–3754 (2021). 10.1007/s10853-020-05461-1

[29] P. Bellet , M. Gasparotto , S. Pressi , A. Fortunato , G. Scapin , M. Mba *et al.*, “ Graphene-based scaffolds for regenerative medicine,” Nanomaterials 11, 404 (2021). 10.3390/nano11020404 33562559 PMC7914745

[30] A. Savchenko , R. T. Yin , D. Kireev , I. R. Efimov , and E. Molokanova , “ Graphene-based scaffolds: Fundamentals and applications for cardiovascular tissue engineering,” Front. Bioeng. Biotechnol. 9, 797340 (2021). 10.3389/fbioe.2021.797340 34950649 PMC8688816

[31] L. Feng , W. Li , J. Ren , and X. Qu , “ Electrochemically and DNA-triggered cell release from ferrocene/β-cyclodextrin and aptamer modified dualfunctionalized graphene substrate,” Nano Res. 8, 887–899 (2015). 10.1007/s12274-014-0570-4

[32] H. J. Yoon , A. Shanker , Y. Wang , M. Kozminsky , Q. Jin , N. Palanisamy *et al.*, “ Tunable thermal-sensitive polymer-graphene oxide composite for efficient capture and release of viable circulating tumor cells,” Adv. Mater. 28, 4891–4897 (2016). 10.1002/adma.201600658 27115557 PMC5680542

[33] Z. Dai , Y. Wang , L. Liu , X. Liu , P. Tan , Z. Xu *et al.*, “ Hierarchical graphene‐based films with dynamic self‐stiffening for biomimetic artificial muscle,” Adv. Funct. Mater. 26, 7003–7010 (2016). 10.1002/adfm.201503917

[34] W. Li , J. Wang , J. Ren , and X. Qu , “ 3D graphene oxide-polymer hydrogel: Near-infrared light-triggered active scaffold for reversible cell capture and on-demand release,” Adv. Mater. 25, 6737–6743 (2013). 10.1002/adma.201302810 24123218

[35] W. Li , J. Wang , J. Ren , and X. Qu , “ Near-infrared upconversion controls photocaged cell adhesion,” J. Am. Chem. Soc. 136, 2248–2251 (2014). 10.1021/ja412364m 24467474

[36] N. Mauro , S. E. Drago , G. Cavallaro , and G. Giammona , “ Near-infrared, light-triggered, on-demand anti-inflammatories and antibiotics release by graphene oxide/elecrospun PCL patch for wound healing,” C—Journal of Carbon Research. 5(4), 63 (2019). 10.3390/c5040063

[37] L. Ferroni , C. Gardin , F. Rigoni , E. Balliana , F. Zanotti , M. Scatto *et al.*, “ The impact of graphene oxide on polycaprolactone PCL surfaces: Antimicrobial activity and osteogenic differentiation of mesenchymal stem cell,” Coatings 12, 799 (2022). 10.3390/coatings12060799

[38] S. Yıldırım , T. T. Demirtaş , C. A. Dinçer , N. Yıldız , and A. Karakeçili , “ Preparation of polycaprolactone/graphene oxide scaffolds: A green route combining supercritial CO_2_ technology and porogen leaching,” J. Supercrit. Fluids 133, 156–162 (2018). 10.1016/j.supflu.2017.10.009

[39] Y. Li , C. Liao , and S. C. Tjong , “ Synthetic biodegradable aliphatic polyester nanocomposites reinforced with nanohydroxyapatite and/or graphene oxide for bone tissue engineering applications,” Nanomaterials 9, 590 (2019). 10.3390/nano9040590 30974820 PMC6523566

[40] B. M. Bijonowski , *Spatiotemporal Regulation of Cell–Cell Adhesions*, edited by M. Anwar , Z. Farooq , R. A. Rather , M. Tauseef , and T. Heinbockel ( IntechOpen, Rijeka, 2021).

[41] A. Seyedsalehi , L. Daneshmandi , M. Barajaa , J. Riordan , and C. T. Laurencin , “ Fabrication and characterization of mechanically competent 3D printed polycaprolactone-reduced graphene oxide scaffolds,” Sci. Rep. 10, 22210 (2020). 10.1038/s41598-020-78977-w 33335152 PMC7747749

[42] P. Haji Mohammadi Gohari , M. Haghbin Nazarpak , and M. Solati-Hashjin , “ The effect of adding reduced graphene oxide to electrospun polycaprolactone scaffolds on MG-63 cells activity,” Mater. Today Commun. 27, 102287 (2021). 10.1016/j.mtcomm.2021.102287

[43] W. Wang , J.-X. Chen , Y. Hou , P. Bartolo , and W.-H. Chiang , “ Investigations of graphene and nitrogen-doped graphene enhanced polycaprolactone 3D scaffolds for bone tissue engineering,” Nanomaterials 11, 929 (2021). 10.3390/nano11040929 33917418 PMC8067503

[44] P. R. Lopes Nalesso , W. Wang , Y. Hou , L. Bagne , A. T. Pereira , J. V. Helaehil *et al.*, “ *In vivo* investigation of 3D printed polycaprolactone/graphene electro-active bone scaffolds,” Bioprinting 24, e00164 (2021). 10.1016/j.bprint.2021.e00164

[45] C. Zhang , D. M. Dabbs , L.-M. Liu , I. A. Aksay , R. Car , and A. Selloni , “ Combined effects of functional groups, lattice defects, and edges in the infrared spectra of graphene oxide,” J. Phys. Chem. C 119, 18167–18176 (2015). 10.1021/acs.jpcc.5b02727

[46] S. Guo , S. Garaj , A. Bianco , and C. Ménard-Moyon , “ Controlling covalent chemistry on graphene oxide,” Nat. Rev. Phys. 4, 247–262 (2022). 10.1038/s42254-022-00422-w

[47] F. Amato , A. Motta , L. Giaccari , R. Pasquale , F. Scaramuzzo , R. Zanoni *et al.*, “ One-pot carboxyl enrichment fosters water-dispersibility of reduced graphene oxide: A combined experimental and theoretical assessment,” Nanoscale 5, 893 (2023). 10.1039/D2NA00771A PMC9890975 36756527

[48] T. Elzein , M. Nasser-Eddine , C. Delaite , S. Bistac , and P. Dumas , “ FTIR study of polycaprolactone chain organization at interfaces,” J. Colloid Interface Sci. 273, 381–387 (2004). 10.1016/j.jcis.2004.02.001 15082371

[49] O. Hartman , C. Zhang , E. L. Adams , M. C. Farach-Carson , N. J. Petrelli , B. D. Chase *et al.*, “ Biofunctionalization of electrospun PCL-based scaffolds with perlecan domain IV peptide to create a 3-D pharmacokinetic cancer model,” Biomaterials 31, 5700–5718 (2010). 10.1016/j.biomaterials.2010.03.017 20417554 PMC2875366

[50] M. Ermeydan , E. Cabane , P. Hass , J. Koetz , and I. Burgert , “ Fully biodegradable modification of wood for improvement of dimensional stability and water absorption properties by poly(ε-caprolactone) grafting into the cell walls,” Green Chem. 16, 3313–3321 (2014). 10.1039/c4gc00194j

[51] A. Baranowska-Korczyc , A. Warowicka , M. Jasiurkowska-Delaporte , B. Grześkowiak , M. Jarek , B. Maciejewska *et al.*, “ Antimicrobial electrospun poly ε-caprolactone scaffolds for gingival fibroblast growth,” RSC Adv. 6, 19647 (2016). 10.1039/C6RA02486F

[52] P. Taddei , A. Tinti , and G. Fini , “ Vibrational spectroscopy of polymeric biomaterials,” J. Raman Spectrosc. 32, 619–629 (2001). 10.1002/jrs.723

[53] V. Palmieri , F. Amato , A. G. Marrani , G. Friggeri , G. Perini , A. Augello *et al.*, “ Graphene oxide-mediated copper reduction allows comparative evaluation of oxygenated reactive residues exposure on the materials surface in a simple one-step method,” Appl. Surf. Sci. 615, 156315 (2023). 10.1016/j.apsusc.2022.156315

[54] W. Kai , Y. Hirota , L. Hua , and Y. Inoue , “ Thermal and mechanical properties of a poly(ε-caprolactone)/graphite oxide composite,” J. Appl. Polym. Sci. 107, 1395–1400 (2008). 10.1002/app.27210

[55] C. Wan and B. Chen , “ Poly(ε-caprolactone)/graphene oxide biocomposites: Mechanical properties and bioactivity,” Biomed. Mater. 6, 55010 (2011). 10.1088/1748-6041/6/5/055010 21921319

[56] N. Akhigan , N. Najmoddin , H. Azizi , and M. Mohammadi , “ Zinc oxide surface-functionalized PCL/graphene oxide scaffold: Enhanced mechanical and antibacterial properties,” Int. J. Polym. Mater. Polym. Biomater. 72, 1423–1433 (2023). 10.1080/00914037.2022.2100373

[57] V. Palmieri , M. D. Spirito , and M. Papi , “ Graphene-based scaffolds for tissue engineering and photothermal therapy,” Nanomedicine 15, 1411–1417 (2020). 10.2217/nnm-2020-0050 32508272

[58] R. Dwivedi , S. Kumar , R. Pandey , A. Mahajan , D. Nandana , D. S. Katti *et al.*, “ Polycaprolactone as biomaterial for bone scaffolds: Review of literature,” J. Oral Biol. Craniofacial Res. 10, 381–388 (2020). 10.1016/j.jobcr.2019.10.003 PMC6854079 31754598

[59] J. M. Unagolla and A. C. Jayasuriya , “ Enhanced cell functions on graphene oxide incorporated 3D printed polycaprolactone scaffolds,” Mater. Sci. Eng. C 102, 1–11 (2019). 10.1016/j.msec.2019.04.026 PMC6546300 31146979

[60] S. F. Melo , S. C. Neves , A. T. Pereira , I. Borges , P. L. Granja , F. D. Magalhães *et al.*, “ Incorporation of graphene oxide into poly(ɛ-caprolactone) 3D printed fibrous scaffolds improves their antimicrobial properties,” Mater. Sci. Eng. C 109, 110537 (2020). 10.1016/j.msec.2019.110537 32228892

[61] V. Palmieri , F. Bugli , M. C. Lauriola , M. Cacaci , R. Torelli , G. Ciasca *et al.*, “ Bacteria meet graphene: Modulation of graphene oxide nanosheet interaction with human pathogens for effective antimicrobial therapy,” ACS Biomater. Sci. Eng. 3, 619–627 (2017). 10.1021/acsbiomaterials.6b00812 33429629

[62] F. De Maio , V. Palmieri , A. Salustri , G. Perini , M. Sanguinetti , M. De Spirito *et al.*, “ Graphene oxide prevents mycobacteria entry in macrophages through extracellular entrapment,” Nanoscale Adv. 1, 1421 (2019). 10.1039/C8NA00413G 36132595 PMC9419007

[63] M. Papi , V. Palmieri , F. Bugli , M. De Spirito , M. Sanguinetti , C. Ciancico *et al.*, “ Biomimetic antimicrobial cloak by graphene-oxide agar hydrogel,” Sci. Rep. 6, 12 (2016). 10.1038/s41598-016-0010-7 28442744 PMC5431354

[64] V. Palmieri , M. Barba , L. Di Pietro , S. Gentilini , M. C. Braidotti , C. Ciancico *et al.*, “ Reduction and shaping of graphene-oxide by laser-printing for controlled bone tissue regeneration and bacterial killing,” 2D Mater. 5, 15027 (2017). 10.1088/2053-1583/aa9ca7

[65] V. Palmieri , W. Lattanzi , G. Perini , A. Augello , M. Papi , and M. De Spirito , “ 3D-printed graphene for bone reconstruction,” 2D Mater. 7, 022004 (2020). 10.1088/2053-1583/ab6a5d

[66] F. De Maio , E. Rosa , G. Perini , A. Augello , B. Niccolini , F. Ciaiola *et al.*, “ 3D-printed graphene polylactic acid devices resistant to SARS-CoV-2: Sunlight-mediated sterilization of additive manufactured objects,” Carbon 194, 34–41 (2022). 10.1016/j.carbon.2022.03.036 35313599 PMC8926154

[67] R. Chalise , A. Niroula , P. Shrestha , B. Paudel , D. Subedi , and R. Khanal , “ A low-cost goniometer for contact angle measurements using drop image analysis: Development and validation,” AIP Adv. 13, 85123 (2023). 10.1063/5.0164668

[68] A. Rosenkranz , G. Perini , J. Y. Aguilar-Hurtado , D. F. Zambrano , B. Wang , B. Niccolini *et al.*, “ Laser-mediated antibacterial effects of few- and multi-layer Ti_3_C_2_T_x_ MXenes,” Appl. Surf. Sci. 567, 150795 (2021). 10.1016/j.apsusc.2021.150795

[69] S. G. Colombarolli , A. Vitali , and F. Sciandra , *Extracellular Vesicle Molecular Profiling for Diagnostic Purposes: An Application of Phage Display Technology BT - Peptide Microarrays: Methods and Protocols*, edited by M. Cretich and A. Gori ( Springer US, New York, 2023), pp. 237–247. 10.1007/978-1-0716-2732-7_17 36152292

